# Annotations

**Published:** 1900-02-10

**Authors:** 


### Annotations.

### Fashion in Nomenclature.

A curious instance of the effect of fashion in modifying the apparent prevalence of certain diseases has come to light in the report of a committee appointed by the Royal College of Physicians to consider the certification and classification of deaths from diarrhœa. The report states that many different terms are employed to designate the disease known as "epidemic diarrhœa," the result being that its specific character is in danger of being ignored, and great statistical confusion is produced. A statement furnished by Dr. Tatham shows the impracticability of picking out from the long list of fashionable but unauthorised synonyms all the cases which in their nature are indistinguishable from diarrhœa, and ought to be called by the authorised name. Take, for example, the returns for diarrhœa and for enteritis. They tell us that the different counties in England vary very widely in the proportion of deaths from these two diseases. But the truth seems to be that this variation is due to mere local fashion; what is called diarrhœa in one place being named enteritis in another, and *vice versâ*. So in London during the quarter ending with September 3,798 deaths were registered as due to diarrhœa; but in addition there were 2,289 deaths from enteritis, and of these 1,880 were certified to be from gastro-enteritis. Mere fashion! There can be no doubt that the bulk of all these cases were to be attributed to epidemic diarrhœa. Diarrhœa, however, is a common name; moreover it is regarded as a trivial ailment, and one from which a clever doctor should hardly let his patient die. Hence we find these deaths returned as dysentery, dysenteric diarrhœa, intestinal catarrh, gastro-intestinal or gastro-enteric catarrh, or more simply as gastro-enteritis, muco-enteritis, or gastric catarrh. Thus the doctor saves his reputation at the expense of the statistics, and although he no doubt tells the truth, the fashionable and fancy synonyms which he uses unfortunately do not tell the whole truth, for they slur over the zymotic nature of the disease. The committee recommended that the college should authorise the use of the term "epidemic," or, if preferred by the practitioner, "zymotic enteritis," as a synonym for epidemic diarrhœa; a pandering to fashion perhaps, but still not without utility. What certainly will be useful is the further recommendation that the college should urge the entire disuse of such terms as "gastro-enteritis," "muco-enteritis," or "gastric catarrh" in cases of epidemic diarrhœa. The whole affair shows how weak is our system of notification, and how the intrusion of a dreaded term such as the word "epidemic" may influence the returns which form the very basis on which statistics are built up.

### Influenza and High Death-rates.

People are apt to become alarmed when such a wave of high death-rates as we have recently experienced passes over the country. Truly, the rates reached in some towns, under the influence partly of inclement weather and partly of widely prevalent influenza, have been very startling. When Brighton loses its inhabitants at the rate of 44·9 per 1,000 per annum, London at the rate of 37·1, Preston of 42·5, and Nottingham of 44·9, it is evident that some very abnormal and evil influence is at work. But it is worth remembering that such death-rates, coming once in a way, do not of necessity say anything against the health and general salubrity of the towns in question. Some very salubrious towns in which the average span of life is long have, month after month, an apparently fabulously low death-rate. Watering-places are fond of advertising their death-rates as running at about 8 or 9 per 1,000, which they no doubt do for long stretches at a time. But the average has to be made up sooner or later, and broadly, it may be said that the death-rate of any place which from its average salubrity is well supplied with old inhabitants, is liable to run up to a high figure when cold winds blow and influenza is at large. Such abnormal rates, however, quickly cure themselves, the feeble folk fall before the storm, and the rate comes down again, as, for example, has now happened at Brighton, where in three weeks the death-rate fell from 44·9 to 17·2 per 1,000. Quite otherwise is it when these high rates persist. When we hear, as we now do, of the average mortality being 43·96 at Dublin, and 51·74 at Cork for five weeks in succession we may be sure that there is something seriously wrong.

### The Housing of the Working Classes.

At a recent meeting of the Council of the Charity Organisation Society, Mr. C. S. Loch, secretary of the society, read a paper on the need for a new inquiry as to the housing of the working classes. He pointed out that, especially in the central districts, the evil of overcrowding, both in the home and on the area, was at present being aggravated rather than ameliorated by the action of the County Council; and he urged the desirability of promoting measures for decreasing the population of the central districts in the interests of the great mass of the people. He reviewed the financial problem created by the entry of the County Council into the trade of house builders, and he showed that not only was the resulting burden on the rates likely to be considerable, but that, even if such trading were remunerative in the long run, it would probably only add to the difficulties of the problem it was trying to solve, by adding yet another competitor for the property required for residential purposes in Central London. Finally, he advocated the establishment of a system of electric tramways converging to a circle round the middle of London, so as to leave the crowded central streets free from such traffic, while making it easy to come and go from them. He very truly said that London was far behind the chief cities of Europe in the application of electricity to tramways. It is, indeed, a constant source of astonishment to us that public bodies should go on adding to the admitted evils of overcrowding on the area by using the rates for house building on central sites, instead of using their own streets, of which they are absolute masters, for promoting transit in a better and more humane manner than by omnibuses and horse-trams. There are millions of pounds waiting for investment in the building of houses for the working classes if the County Council would but give some assurance that the rates would be spent in promoting locomotion rather than in competing in the trade of house building. Mr. Loch's remarks deserve the most careful consideration.

